# Disability trajectories prior to death for ten leading causes of death among middle-aged and older adults in Taiwan

**DOI:** 10.1186/s12877-021-02300-z

**Published:** 2021-07-10

**Authors:** Ching-Ju Chiu, Meng-Ling Li, Chia-Ming Chang, Chih-Hsing Wu, Maw Pin Tan

**Affiliations:** 1 Institute of Gerontology, College of Medicine, National Cheng Kung University, No. 1 University Road, 701 Tainan, Taiwan; 2 Department of Medicine, College of Medicine, National Cheng Kung University, Tainan, Taiwan; 3 Division of Geriatrics and Gerontology, Department of Internal Medicine, National Cheng Kung University Hospital, Tainan City, Taiwan; 4 Department of Family medicine, College of Medicine, National Cheng Kung University Hospital, National Cheng Kung University, Tainan, Taiwan; 5 Department of Medicine, Faculty of Medicine, University of Malaya, Kuala Lumpur, Malaysia

**Keywords:** ten leading causes of death, functional impairment, disability trajectory, longitudinal study, Asia

## Abstract

**Background:**

Prolonged life expectancy is associated with increased prevalence of chronic diseases. The aim of this study was to determine the different disability trajectories for the top ten leading causes of death in Taiwan .

**Methods:**

A total of 2,431 participants aged 50–96 in 1996 from the Taiwan longitudinal study on aging (TLSA) who died from 1996 to 2016 were analyzed. Integration of Cause of Death Data and TLSA helped sort out participants who had died from the ten leading causes of death. The level of physical disability was evaluated with the Activities of Daily Living Scale (ADLs), ranging from 0 to 6 points, in 1996, 1999, 2003, 2007, and 2011. A multilevel model was used to investigate the levels and rates of change in disability development before death.

**Results:**

The outcome of the research showed that the earliest group to experience physical limitation was individuals living with diabetes. The groups with the highest ADL scores were participants with diabetes, cerebrovascular disease, and hypertension-related diseases. Most groups reach ADL scores ≥ 1 (mild-level) during 4–6 years before death except chronic hepatitis and cirrhosis and injury.

**Conclusions:**

People who had died from the ten leading causes of death experienced different disability trajectories before death. The trajectory of the participants who had died from diabetes showed a unique pattern with the earliest occurrence and more severe deterioration in terms of development of disabilities. Disability trajectories provide a prediction of survival status for middle-aged and older adults associated with the ten leading causes of death.

## Background

Chronic diseases dominate the top 10 leading causes of death among people aged over 65 years in Taiwan, including cancer, heart disease, cerebrovascular conditions, diabetes, chronic lung disease, hypertensive disease, and renal diseases [1]. National health statistics show that 88 % of older Taiwanese individuals have one or more chronic diseases [2]. In population aged over 65 in Taiwan, the prevalence of activities of daily living (ADL) was 12–14 % [3]. Chronic diseases are the main cause of poor health, disability, and death, and account for most healthcare expenditures [4]. The Leiden 85-plus studies indicated that older people with chronic diseases, cognitive impairment, and depression are at high risk of further deteriorations in functional ability [5–7]. A systematic analysis of the Global Burden of Disease Study 2010 estimated that the disability-adjusted life years (DALYs) ranked in descending order were ischemic heart disease, followed by lower respiratory infections and stroke [8]. In the 21st century, the proportion of individuals living with disabilities has increased and has become a heavy social burden [9]. There is a gap in the literature, however, on the dynamic changes in the disability trajectories associated with different leading causes of death.

Men and women are different in terms of their body composition (hormone concentration, bone mineral density, muscle mass) and social behavior [10, 11]. Existing studies indicate that men are more susceptible to diseases with a high risk of mortality like cardiovascular diseases, respiratory disease, and accidents, while women are more vulnerable to lower risk diseases such as diabetes, musculoskeletal diseases, and anxiety-depression symptoms [12–14]. In ischemic stroke patients, both disability and mortality rates are higher in women than in men and diabetes was an independant predictor of 1-year disability for women [15]. A study calculates the time spent with diseases in remaining life expectancy among Dutch elderly over 65 found the most reduction of disability-free life expectancy was attributed to coronary heart disease in men and osteoarthrtis in women, respectively. Combination of osteoarthritis and diabetes cause the most time spent with disease in both sex. Specific and co-morbidity shape the different life course of men and women, determining the time they spent with disease and their potential disability [16]. Although previous studies have identified gender differences in disability during aging, there is limited information related to the timing of the gender inequality before death associated with various chronic conditions.

As disability is a health burden to be reckoned with, new and effective intervention strategies should be identified [17]. Projections of mortality and disability are useful aids in decision-making related to priorities for health research, capital investment, and training [18]. For example, in terms of policies, this model can be adapted to modify long-term care systems by predicting the number of people who comprise the potential disabled population. Furthermore, such studies help distribute resources to different diseases depending on their severity level. To fill this gap in knowledge, the aim of this study was to determine disability trajectories associated with various leading causes of death. We also examined the patterns by gender.

## Method

### Data sources & participants

Our data were drawn from the Taiwan Longitudinal Study on Aging (TLSA), which was conducted by the Ministry of Health and Welfare in 1989 on a nationally representative sample of adult residents in non-aboriginal townships in Taiwan. This nationally representative survey is aimed toward tracing longitudinal changes in the health, behavioral, financial, and emotional well-being of middle-aged and older adults in Taiwan. During the first wave of sample collection in 1989, a nationally representative sample of 4,412 adults aged 60 years or older was selected, and 4,049 were successfully interviewed (response rate = 92 %). In the second wave of data collection in 1993, 3,467 individuals had survived, and 3,155 completed the survey (response rate = 91 %). In the third wave of data collection in 1996, 3,002 individuals had survived, and 2,699 completed the survey (response rate = 89 %). To replenish the younger part of the respondent population, 2,462 individuals who were born between 1930 and 1945 were interviewed. Thus, a total of 5,131 individuals in the 1996 TLSA were representative of the entire Taiwan population aged 50 years or older living in the community or in institutions.

**Fig. 1:**
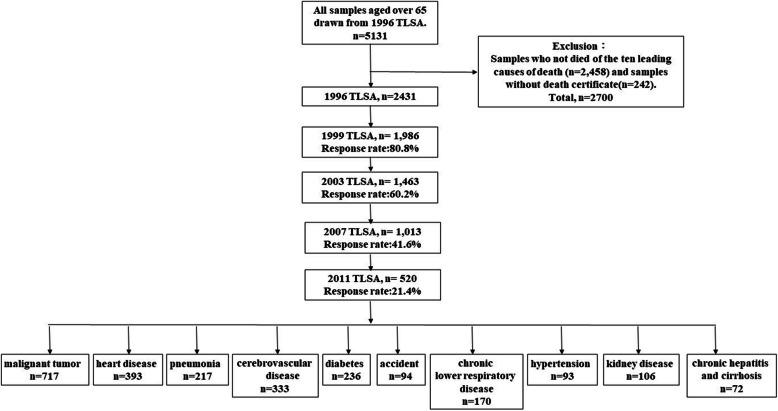
Flow chart of the inclusion of the participants

In this study, the participants were over 50 in the 1996 TLSA as well as in their follow-up records in 1999, 2003, 2007, and 2011. Respondents who died from 1997 to 2016 and died from the ten leading causes of death comprised the final database to be analyzed. A sample of 5,131 adults was selected from the 1996 TLSA, of which 1,427 (27.81 %) were aged between 50 and 65, and 3,704 (72.19 %%) were aged over 65 years. We excluded adults who didn’t die from the ten leading causes of death (N = 2,458) and those not recorded in the death certificate files (N = 242). Five waves of interviews were conducted. In the first wave in 1996, a total of 2,431 participants were included in the baseline data collection. In the second wave in 1999, 1,986 individuals remained from the 1996 data collection (80.8 %). In the third wave in 2003, 1,463 remained from the 1996 data collection (60.2 %). In the fourth wave in 2007, 1,013 remained from the 1996 data collection (41.6 %). In the fifth wave in 2011, 520 remained from the 1996 data collection (21.4 %). Detailed participant inclusion criteria are provided in Fig. 1.

#### Measures

We conducted a retrospective cohort study to examine the different disability trajectories of participants who had died from the ten leading causes of death based on death statistics in Taiwan in 2017, including (1) malignant tumor, (2) heart disease, (3) pneumonia, (4) cerebrovascular disease, (5) diabetes, (6) injury, (7) chronic lower respiratory disease, (8) hypertension-related diseases, (9) kidney disease, and (10) chronic hepatitis and cirrhosis. The cause of death was obtained from death certificate files managed by the Ministry of Health and Welfare. Since the data period ranged from 1997 to 2016, death certificates before 2007 were drawn based on ICD-9. Those after 2008 were drawn based on ICD-10. Appendix provides the detailed ICD codes for every leading cause of death. Specifically, time was coded to reflect the number of years before death, for example, participants who had died in 2005 with interview records reflecting ADL in the 1996, 1999, and 2003 TLSA. Time in 1996 was coded as Time _1996=_-9; time in 1999 was coded as Time _1999=_-6, and time in 2003 was coded as Time _2003=_-2. ADL could thus be traced up to 20 years before death (Time _1996=−_20, died in 2016).

The dependent variable, physical disability, was measured with the modified Katz Activities of Daily Living (ADL) [19]. ADL-related disabilities affect an individual’s direct self-care, including bathing, dressing, eating, walking across a room, getting in/out of bed, and using a toilet independently [20, 21]. According to the questionnaire, respondents who reported that they had difficulty or were unable to do a given task, or that they received help or used equipment when performing the task were coded as having difficulty with the task (1 = yes, 0 = no) [20]. The final scores were the total of 6 items from the ADL limitation questionnaire (range = 0–6). Socio-demographic characteristics including sex and age at baseline were also independent variables of interest, including determining the disability trajectories of different causes of death for females and males.

### Data analysis

The data were analyzed using SAS software version 9.4 (SAS Institute, Inc., Cary, NC). To examine the physical disability trajectories before death, we employed multilevel modeling (MLM) and computed separate models for each causes of death. The MLM equation models are shown as follows:

Level-1 model:

$$ {Y}_{ij}={\pi}_{0j}+{\pi}_{1j}{\left( years\ before\ death\right)}_{ij}+{\pi}_{2j}{{\left( years\ before\ death\right)}^2}_{ij}+{r}_{ij,} $$

where *r*_*ij*_~*N*(0, *σ*^2^).

Level-2 model:

$$ {\pi}_{0j}={\beta}_{00}+{\beta}_{01} Age\  at\ {diagnosis}_j+{u}_{0j}, $$

$$ {\pi}_{1j}={\beta}_{10}+{\beta}_{11} Age\  aat\ {diagnosis}_j+{u}_{1j}, $$

$$ {\pi}_{2j}={\beta}_{10}+{\beta}_{11} Age\  aat\ {diagnosis}_j+{u}_{1j}, $$

where $$ \left(\begin{array}{c}\begin{array}{c}{u}_{oj}\\ {}{u}_{1j}\end{array}\\ {}{u}_{2j}\end{array}\right)\sim N\left[\left(\begin{array}{c}0\\ {}0\\ {}0\end{array}\right),\left(\begin{array}{ccc}{\tau}_{00}& {\tau}_{01}& {\tau}_{02}\\ {}{\tau}_{10}& {\tau}_{11}& {\tau}_{12}\\ {}{\tau}_{20}& {\tau}_{21}& {\tau}_{22}\end{array}\right)\right]. $$

${Y}_{ij}$ was the ADL score of participant j at time i. i=-20 ~ 0. To facilitate interpretation of the ${\pi}_{0j}$intercept, we centered the time to 0. The intercept (time=-20) thus represented 20 years before death. As shown in the Level 2 model, cross-level age and time interaction was estimated to allow the estimation of the intercept, slope, and curvature of the disability trajectory to be adjusted based on the participant age at diagnosis.

## Results

### Participant characteristics and raw mean scores for development of disability on years 1, 5, 10, 15, and 20 before death

As shown in Table 1, the final number of participants meeting our requirements was as follows: malignant tumor (*n* = 717), heart disease (*n* = 393), cerebrovascular disease (*n* = 333), diabetes (*n* = 236), pneumonia (*n* = 217), chronic lower respiratory disease (*n* = 170), kidney disease (*n* = 106), injury (*n* = 94), hypertensive disease (*n* = 93), and chronic hepatitis and cirrhosis (*n* = 72). For the oldest participants, on average, the ten leading causes of death were pneumonia (83.78 ± 8.25), hypertensive disease (83.06 ± 8.12), and chronic lower respiratory disease (82.97 ± 6.77), while malignant tumor (77.51 ± 8.28), injury (76.47 ± 8.58), and chronic hepatitis and cirrhosis (75.26 ± 9.34) comprised the participants with the lowest average age.

**Table 1:** Participant characteristics and raw mean scores of disability development at 1, 5, 10, 15, and 20 years prior to death

	Ten leading causes of death									
	malignant tumor	Heart disease	pneumonia	cerebrovascular disease	diabetes	injury	chronic lower respiratory disease	hypertension-related diseases	kidney disease	chronic hepatitis and cirrhosis
N	717	393	217	333	236	94	170	93	106	72
**age**	77.51±8.28	81.90±8.20	83.78±8.25	80.41±8.15	78.00±5.81	76.47±8.58	82.97±6.77	83.06±8.12	80.39±7.52	75.26±9.34
**Sex**										
**Male, N(%)**	467(65.13)	208(52.93)	147(67.74)	184(55.26)	89(37.71)	66(70.21)	129(75.88)	41(44.09)	49(46.23)	45(62.50)
**Female, N(%)**	250(34.87)	185(47.07)	70(32.26)	149(44.74)	147(62.29)	28(29.79)	41(24.12)	52(55.91)	57(53.77)	27(37.50)
**ADL(0-6)Raw data**										
**1**^**st**^**year before death**	1.06±2.04	1.62±2.43	2.81±2.72	3.25±2.86	3.07±2.77	0.31±1.19	2.03±2.48	2.90±2.87	2.48±2.71	0.69±1.74
**5**^**th**^**year before death**	0.27±1.07	0.86±1.80	0.77±1.59	1.28±2.32	1.41±2.38	0.17±0.82	0.76±1.83	1.07±2.07	1.64±2.36	0.73±1.85
**10**^**th**^**year before death**	0.05±0.37	0.35±1.23	0.26±1.12	0.07±0.45	0.50±1.35	0.43±1.60	0.88±2.09	0±0 (0-0)	0.56±1.38	0±0
**15**^**th**^**year before death**	0±0	0±0	0.39±1.37	0.29±1.08	0.45±1.28	0±0	0±0	0±0	0±0	0±0
**20**^**th**^**year before death**	0±0	0±0	0±0	0±0	0±0	0±0	0±0	0±0	0±0	0±0

Note: The timing of the ADL scores was coded to reflect the number of years before death (-20~0). In this table, we centered time to 0.The intercept (time=-20) thus represents 20 years before death

The distribution of males and females was also different across different causes of death. The proportion of females with specific diseases surpassing males included diabetes (female: 62.3 %), hypertensive disease (female: 55.9 %), and kidney disease (female: 53.8 %). Male participants dominated in cause of death from chronic lower respiratory disease (male: 75.9 %), injury (male: 70.2 %), pneumonia (male: 67.7 %), malignant tumor (male: 65.1 %), chronic hepatitis and cirrhosis (male: 62.5 %), cerebrovascular disease (male: 55.3 %), and heart disease (male: 52.9 %).

The raw mean ADL limitation scores for the top ten leading causes of death are illustrated in Table 1. To help with clinical observation, the 1st, 5th, 10th, 15th, and 20th years prior to death are also shown in Table 1. Chronologically, it can be observed that in the 20th year before death, the mean ADL limitation score was 0 for all participants. However, in the 15th year before death, participants subsequently dying from pneumonia, cerebrovascular disease, or diabetes dominated in terms of physical disabilities, with participants dying from diabetes experiencing the highest levels of disability the 15th year prior to death. The 10th year before death, with the exception of hypertension-related disease and chronic hepatitis and cirrhosis, participants dying from the other eight diseases reported some physical disabilities. Participants dying from diabetes or chronic lower respiratory disease reported ADL limitation scores averaging 0.88 ± 2.09 and 0.50, respectively. In the 5th year before death, participants in all ten categories suffered from physical disabilities. Participants who died from kidney disease (ADL = 1.64 ± 2.36), diabetes (ADL = 1.41 ± 2.38), cerebrovascular disease (ADL = 1.28 ± 2.32), and hypertension-related diseases (ADL = 1.07 ± 2.07) reported an average of at least one ADL limitation. As for the very last year before death, the ADL limitation scores of participants dying from cerebrovascular disease, diabetes, and hypertension-related diseases sharply increased, with an average of 3.25, 3.07, and 2.90 limitations in ADL, respectively.

### Disability trajectories for the ten leading causes of death

As shown in Table 2, the samples were analyzed using two-level multilevel modeling: ADL limitation score _tdx(i)_ = intercept_tdx(i)_ + β_1(i)_*time_tdx(i)_ + β_2(i)_*time^2^_tdx(i)_. ( Intercept: the baseline ADL score in the HLM model; time: increases in disability every year approaching death; time^2^ accelerated rate of disability every year approaching death).

**Table 2:** Fixed effects coefficient estimate of disability trajectories for the ten leading causes of death by sex

Fixed effects coefficients	malignant tumor	heart disease	pneumonia	cerebrovascular disease	diabetes	Injury	chronic lower respiratory disease	hypertension-related diseases	kidney disease	chronic hepatitis and cirrhosis
**Entire sample**										
**Intercept**	0.0976*	0.2681*	0.7242***	0.9776***	0.8456***	-0.0507	0.6413**	0.2072	0.4535	0.0034
**Time**	-0.0342*	-0.0513*	-0.0146	-0.0697	0.0015	0.0153	-0.0389	-0.1221**	0.0097	0.0286
**Time2**	0.0085***	0.0156***	0.0164***	0.0176***	0.0130**	-0.0005	0.0135***	0.0256***	0.0127*	0.0087
**Intercept**	0.8832***	1.4722***	2.4321***	3.0680***	1.9713***	0.4717*	2.0233***	2.3369***	2.0129***	0.9210*
**Time**	0.1358***	0.1819***	0.3470***	0.4848***	0.2821**	0.05898	0.3283***	0.3690***	0.3001**	0.08661
**Time2**	0.0051***	0.0057	0.0121***	0.0187***	0.01016*	0.0019	0.0124***	0.0136**	0.0110*	0.0021
**Intercept**	1.1013***	2.4063***	3.9302***	3.4569***	3.3168***	0.6238*	2.6899***	3.4208***	2.8997***	0.9828*
**Time**	0.1567***	0.3104***	0.5280***	0.3889***	0.3711***	0.08595	0.2616	0.5668***	0.3794**	0.1109
**Time2**	0.0055**	0.0100***	0.0185**	0.0112**	0.0113**	0.0028	0.0070	0.0235***	0.0124*	0.00310

Note: All coefficients were controlled for the age at which death occurred. ***p < 0.001;**p < 0.01;* *p*< 0.05

**Fig. 2:**
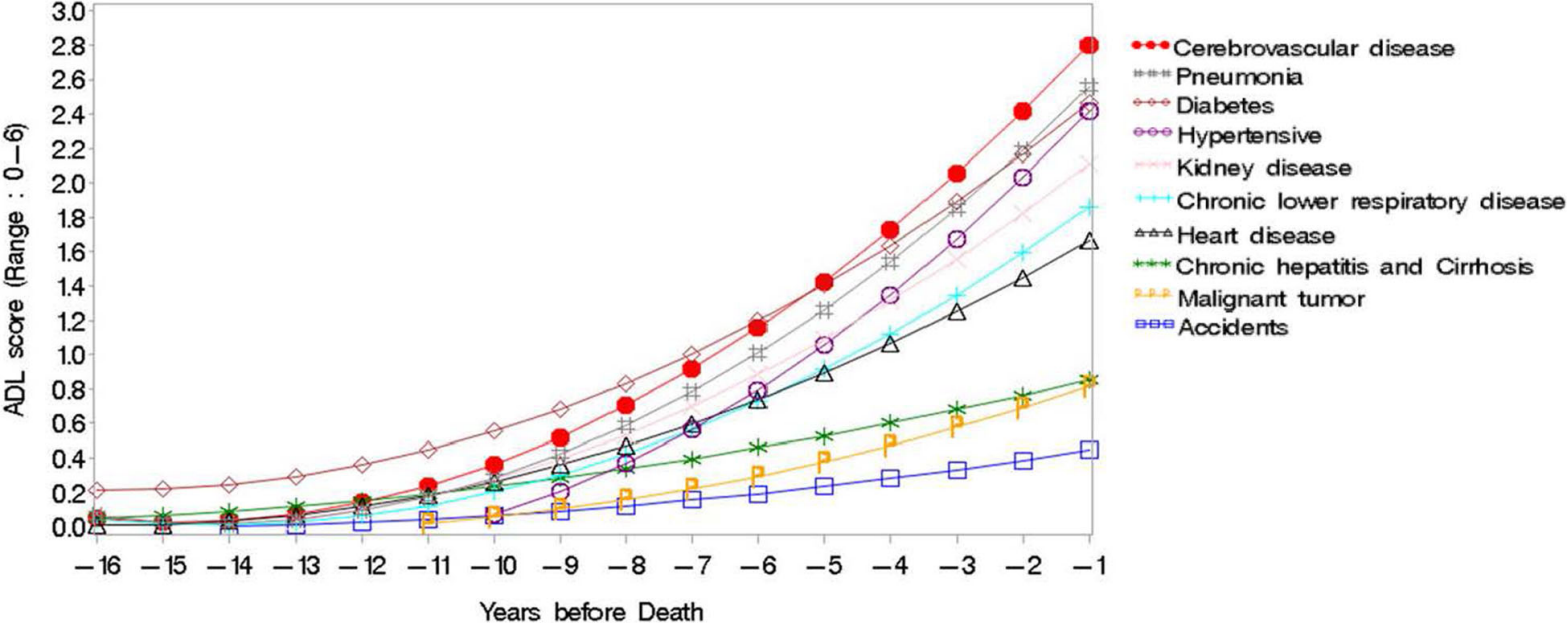
Disability development for descendants dying from the leading causes of death

Figure 2 illustrates the different disability trajectories for the ten leading causes of death predicted using the multilevel model. With the illustration, the magnitude and turning points of disability development across each cause of death can be easily understood. First, cerebrovascular disease (3.25 ± 2.86), pneumonia (2.81 ± 2.72), and diabetes (3.07 ± 2.77) were the three diseases with the highest ADL limitations in the last year of life. By contrast, injury (0.31 ± 1.19), malignant tumor (1.06 ± 2.04), and chronic lower respiratory disease (2.03 ± 2.48) were the categories with the lowest ADL limitations. Second, observing the slope of these curves, it can be seen that hypertension-related diseases, cerebrovascular disease, and pneumonia exhibited incremental increases in ADL limitations toward the end of life. Participants who died of these diseases experienced more dramatic worsening of disabilities before their end of life. Based on the definition of the severity of physical dysfunction, we divided the disability score into three standards: mild (ADL limitation scores = 1), moderate (ADL limitation scores = 2), and severe (ADL limitation scores > 3) [16]. During the last year of life before death, cerebrovascular disease, pneumonia, diabetes, hypertension disease-related diseases, and kidney disease were classified at the “moderate” level of disability, while chronic lower respiratory disease and heart disease lay between mild and moderate levels of disability. The rise in disability levels on every curve began 13 years prior to death. Most groups had ADL limitation scores of more than one, at the mild level, 4–6 years before death with the exception of chronic hepatitis, cirrhosis, and injury, where participants dying from these diseases showed a gentle incremental rise in ADL limitations, for which the ADL scores were below the mild level. However, there was an outstanding diabetes curve for which the starting point was different from the others in terms of an earlier starting point and a longer period of disability.

## Discussion

Using trajectory modeling, the longitudinal changes in ADL scores for 2,431 decedents dying from the ten leading causes of death in 20 years were established. The disability trajectories associated with the ten conditions were dynamic with regards to the overall disability level. Specifically, some individuals with such as diabetes, pneumonia, and cerebrovascular disease were confronted with disabilities earlier and more severe than the other diseases.

A systematic analysis for the Global Burden of Disease Study 2019 revealed that, in both the 50–74-year and 75-years-and-older age groups, ischaemic heart disease and stroke were the main cause of disability-adjusted life-years (DALYs), especially in low or middle-income country [17]. However, the present study found that heart diseases was not the predominant cause of death leading to disability in Taiwan. In our study, for people died of heart diseases, the mean ADL score before death was the fourth to last. There were two plausible explanation for this disparity. First, accessibility rate of cardiology services and median percentages of interventional and electrophysiologists among cardiologists were higher in Taiwan than European countries. Indicating that patients with heart diseases have more procedure capabilities with more alternatives [22]. Second, in comparison to Sweden and America, physicians in Taiwan were more likely to certify diabetes as the underlying causes of death when diabetes and cardiovascular diseases coexisted [36]. It should be noted, however, that our study did not use multiple cause-of-death statistics which would be mentioned at the limitation parts.

An average of one limitation in ADL during the four to six years before death was found in the present study, except chronic hepatitis, cirrhosis, and injury for which there were no apparent disabilities during that period. Compared to previous studies on disability trajectories, the results show some similarities and differences in terms of the worsening trend of disability in late life with a transition from no disability to greater disability and death. Three distinct illness trajectories were described for people with chronic conditions, including (1) a short period of evident decline, typically cancer, (2) long term limitations with intermittent serious episodes, for example, respiratory and heart failure, and (3) prolonged gradual decline (typical of frail elderly people or people with dementia) [23–25]. There was some disparities in the disability trajectories of malignant tumor. The health status and physical impairment of cancer was stable in the previous years after diagnosis. Other studies showed a drastic decline in the last year or last months, but our finding was steadily worsening [26].

The distribution of males and females was also different across ten leading causes of death. Men were more likely to suffer from chronic lower respiratory disease, injury, pneumonia, malignant tumor, chronic hepatitis and cirrhosis, cerebrovascular disease, and heart disease. While women were more vulnerable to diseases like diabetes, hypertensive disease, and kidney disease. Gender differences can be discussed in body composition and social behavior, separately. Women are benefit from hormone-protective effect: estrogen reduces the risk of cardiovascular diseases(CVD) for premenopausal females, and prevents CVD for post-menopause women with early hormone treatment [27]. Besides, estrogen possesses anti-inflammation effect on inhibition of hepatocellular carcinoma (HCC) development and neuroprotective effect on slowing down the progression of injury and suppressing apoptotic pathways in brain [28–30]. In the aspect of social behavior, the prevalence of smoking and alcohol consumption were much higher in men than women, which were the risk factors for many chronic diseases, including cancer, CVD, liver diseases, and neurocognitive disorders [31, 32]. The life expectancy of women with diabetes were longer than men with diabetes; however, women had to spent greater proportion of their life with disability [33]. There are several reasons why women are more vulnerable to kidney diseases. First, for women with complicated hypertensive pregnancies, the risk of developing end-stage renal diseases (ESRD) increases [34]. Second, women are high risk group for autoimmune diseases such as systemic lupus erythematosus, a multisystem disease with high degree of renal involvement, which is likely to progress lupus nephritis or ESRD [35]. For patients with ESRD, women have more complication on dialysis and transplantation, and they are more inclined to conservative care rather than kidney transplantation compared with men [36].

In our study, diabetes was the cause of death associated with the earliest onset of disability and longest course of disability. Diabetes is a complicated chronic disease associated with both physical and mental illness leading to disability. A meta-analysis showed that people with diabetes possessed 50–80 % higher risk of disability comparing with people without diabetes [37]. A previous analysis supports our findings, highlighting the increasingly role of diabetes as a cause of disability at those ranging in age from 50 to 64 [38]. Early-onset disability increased the risk of premature death, prolonged lifespan with disability and impaired life quality [39] Hyperglycemia in individuals with diabetes results in general weakness, blurred vision, and impairment in lower-extremity physical function, which are key contributors to loss of physical independence [40]. Another study indicated that type 2 diabetes is associated with accelerated loss of leg muscle strength and quality [41]. Furthermore, the prevalence of hypertension was higher in people with diabetes [42]. Diabetes increase the stiffness of the arteries, which results in the age-related elevation in systolic blood pressure in comparison to people without diabetes [43, 44]. The risks of microvasular diseases (diabetic nephropathy, neuropathy, and retinopathy) and macrovascular comorbidities (coronary artery disease, peripheral arterial disease, and stroke) complications increase in patient with diabetes and hypertension [45]. These findings externally validate our data and explain why diabetes shapes the distinct disability trajectory with the earliest onset and longest process.

The average age of those who died of pneumonia was higher than the other nine top 10 causes of death. Pneumoniae showed early onset of disability second to diabetes. Pneumonia is a common community-acquired disease that leads to morbidity and mortality in the older population. Older persons are more likely to develop cerebrovascular disease or degenerative neurologic disease, which impairs the “cough reflex” and causes oropharyngeal aspiration. Several pulmonary diseases may occur after aspiration, including pneumonia [46]. In Davydow et al., survivors of hospitalization for pneumonia were at greater risk for ADL and IADL impairments [47]. However, in Salive et al., both mild to moderate and severe impairments were associated with increased risk for pneumonia-related death [48]. The bidirectional relationship between disability and pneumonia explains the earlier onset in disability associated with death from pneumonia compared to the other causes of death, apart from diabetes.

Malignant tumor is the leading cause of death with highest prevalence in Taiwan and has become the major morbidity and cause of mortality globally. In previous studies, it was found that patients with cancer experience sharp functional declines categorized as catastrophic disability in their last year of life [26, 49]. However, in the present study, both the raw and predicted ADL scores for malignant tumor were lower than those for the other leading causes of death, and the slope of the growth curve model was gentle, with the lowest ADL score second to injury in the last year of life. The different outcome can be attributed to the interval and frequency of data collection. In former studies, the participants were interviewed to determine their level of disability in the final years of their life. In the present study, participants in the database were re-interviewed every 3 to 4 years, taking into account that physical function would dramatically deteriorate in cancer patients before death, which might have failed to reflect the true severity of their level of disability.

The strength of the present research is that the samples were derived from a nationally representative longitudinal cohort study recording ongoing changes in physical disability among ten groups over a 20 year period, which made it possible to observe dynamic changes as well as to predict the development of disability long-term. There are some limitations in our study. First, although the World Health Organization (WHO) had defined the underlying cause of death (UCOD) as “the disease or injury which initiated the train of morbid events leading directly to death or the circumstances of the accident or violence which produced the fatal injury” [50]. Nevertheless, physicians might have their own interpretation of UCOD based on different diseases causal relationship, different customs in diagnosing cause of death, and selection rules; thus, the process is difficult to maintain entirely objective [51]. Evaluating the concordance rate between UCOD recorded on death certificate and pathologic report based on autopsy, cancer was relatively higher than cardiovascular diseases and pneumoniae [52]. Take diabetes as an example. The coexistence of microvasular diseases or macrovascular comorbidities and other competing diseases (cancer and choronic obstructive pulmonary diseases) made it more difficult for physicians to determine the UCOD. Thus, we should be aware of the improper diabetes-related cause-of-death statements on death certificates [53, 54]. The accuracy of cause-of-death statistics still had room for improvement. Second, our use of death certificate files to identify a single condition leading to death would not have reflected the presence of other chronic conditions that were likely to co-exist among older population [26]. Underlying causes of death alone was insufficient to fully represent the mortality burden, especially for UCOD such as diabetes mellitus, influenza, pneumonia, chronic renal diseases, and hypertensive diseases [55–57]. The weak point of this study was not providing MCOD statistics at the same time.

In conclusion, given the strengths and limitations of our study, we conclude the following: First, older persons with diabetes may be more likely to experience an earlier and longer disability process. Second, patients with pneumonia and cerebrovascular disease are high-risk populations confronted with severe disabilities at the end of life. Third, malignant tumor, injury, and chronic hepatitis didn’t lead to early disabilities. The disability level of patients with malignant tumors drastically worsens as death approaches. Disability trajectories enable patients and their caregivers to predict the future more accurately and to manage their expectations and plan appropriately. Investigation of disability trajectories prior to death for ten leading causes provides the authorities concerned a target point to intervene. However, medicine is an art intended to better the lives of patients and relieve their discomfort. In addition to discussing disability trajectories passively, perhaps we can look for ways to “reverse” the worsening trend of ADL scores in order to improve quality of life as patients age and offer timely interventions.

## Data Availability

The data that support the findings of this study are available from Health Data Science Center but restrictions apply to the availability of these data, which were used under license for the current study, and so are not publicly available. Data are however available from the authors upon reasonable request and with permission of Health Data Science Center.

## Appendix

**Table 3:** ICD code

	ICD9	ICD10
Malignant tumor	140-208	C00-C97
Heart diseases	390-392,393-398,410-414,420-429	I01-I02.0, I05-I09, I20-I25, I27, I30-I52
Pneumoniae	480-486	J12-J18
Cerebrovascular diseases	430-438	I60-I69
Diabetes	250	E10-E14
Injury	800-949	V01-X59, Y85-Y86
Chronic lower respiratory diseases	490-496	J40-J47
Hypertension-related diseases	401-405	I10-I15
Kidney diseases	580-589	N00-N07, N17-N19, N25-N27
Chronic hepatitis and cirrhosis	571	K70, K73-K74

Death certificates between 1996-2007 were drawn based on ICD-9. Those between 2008-2016 were drawn based on ICD-10

## Abbreviations

ADL: Activities of Daily Living
TLSA: Taiwan Longitudinal Study on Aging
MLM: Multilevel Modeling
